# Idiopathic Hyperprolactinemia Presenting as Polycystic Ovary Syndrome in Identical Twin Sisters: A Case Report and Literature Review

**DOI:** 10.7759/cureus.3004

**Published:** 2018-07-19

**Authors:** Alpesh Goyal, Mohd Ashraf Ganie

**Affiliations:** 1 Endocrinology and Metabolism, All India Institute of Medical Sciences, New Delhi, IND

**Keywords:** hyperprolactinemia, polycystic ovary syndrome, insulin resistance, androgen excess, dopamine agonist, cabergoline, idiopathic hyperprolactinemia

## Abstract

This case report describes 15-year-old identical twin sisters, who presented to us with features of polycystic ovary syndrome (PCOS). A biochemical workup revealed hyperinsulinemia and androgen excess with elevated prolactin levels. The possible etiologies for hyperprolactinemia were excluded with a detailed evaluation and it was labeled as idiopathic. Considering the fact that androgen excess could be caused by either insulin resistance or hyperprolactinemia, we decided to treat one sister with insulin sensitizer metformin and other with dopamine agonist cabergoline. While cabergoline treatment resulted in normalization of prolactin levels and androgen excess, no significant biochemical or clinical improvement occurred with metformin treatment. Hyperprolactinemia was therefore considered to be the cause of androgen excess in both and cabergoline therapy initiated in the other sister as well. Through the report, we conclude that diagnosis of PCOS should be made only after exclusion of alternative causes like hyperprolactinemia and detailed evaluation should be sought for any significant, unexplained prolactin elevation. Although rare, hyperprolactinemia can lead to androgen excess by increasing adrenal androgen secretion, which improves with dopamine agonist therapy.

## Introduction

Prolactin is a 199 amino acid polypeptide hormone produced by the lactotroph cells of anterior pituitary and is under tonic inhibitory control of hypothalamic peptide dopamine. The secretion of prolactin is stimulated by thyrotropin-releasing hormone (TRH), estrogen, epidermal growth factor, vasoactive intestinal peptide (VIP) and dopamine receptor antagonists. Hyperprolactinemia is a state of prolactin excess characterized by symptoms of menstrual irregularities, galactorrhea and rarely hirsutism in females. It may occur secondary to medications, hypothyroidism, renal dysfunction and sellar or parasellar masses. Idiopathic hyperprolactinemia is defined as the presence of persistent high prolactin levels, which cannot be explained by any recognized cause for increased prolactin secretion. Patients with symptomatic hyperprolactinemia due to any cause need treatment, including treatment of underlying condition (as hypothyroidism or stopping offending medication) or use of dopamine agonist in prolactinoma/idiopathic hyperprolactinemia. When treatment with dopamine agonist cabergoline is initiated, the patient should be followed closely for symptoms and prolactin levels, and treatment withdrawal attempted after at least two years of therapy, provided prolactin levels have normalized and no tumor remnant is seen on neuro-imaging [1].

Polycystic ovary syndrome (PCOS) is the most common endocrine disorder affecting women of reproductive age group, with prevalence varying from 4 to 18%, depending on the criteria used [2,3]. The prevalence of PCOS is rising at exponential proportions worldwide, in parallel with the ever increasing obesity and type 2 diabetes mellitus. The exact pathophysiology of this disorder with a host of metabolic, cardiovascular and reproductive implications is not clear, however, both genetic and environmental factors are known to contribute to its causation [4,5]. Insulin resistance (IR) contributes to the pathophysiology of PCOS in 50–80% women, especially those who have severe phenotype and those who are overweight. IR not only leads to the elevated cardio-metabolic risk, but also contributes to reproductive features by increasing androgen production and decreasing sex hormone binding globulin (SHBG). Diagnosis of PCOS is one of exclusion; it is made after excluding alternative causes of androgen excess, such as ovarian or adrenal neoplasm, non-classical congenital adrenal hyperplasia (NCCAH), hypothyroidism, hyperprolactinemia, Cushing’s syndrome (CS) and acromegaly. Management of PCOS requires multidisciplinary approach, and usually varies depending on the predominant symptom. Diet and lifestyle therapy should be the first-line of treatment in all women with PCOS who are overweight/obese. Various studies have shown that even a weight loss of 5–10% can lead to improvement in the reproductive, metabolic and psychological disturbances associated with the disorder [4]. Medical therapy is predominantly directed at treatment of the concerning symptoms and includes insulin sensitizers (such as metformin, thiazolidinediones), oral contraceptives, antiandrogens (such as spironolactone, cyproterone acetate), and clomiphene citrate or gonadotropins for infertility. However, currently there is no ideal therapy that could fully reverse the underlying hormone disturbances and treat all the clinical features associated with this disorder [5].

## Case presentation

Patients T and S, identical twin sisters of age 15 years, presented to us with complaints of progressive weight gain and velvety hyperpigmentation with associated skin thickening over the nape of neck, axilla and front of elbow over the past 4–5 years. Both the girls had generalized weight gain, which was not associated with any history of growth failure, red striae, proximal myopathy or easy bruising to suggest CS. A dietary history revealed that both sisters had faulty eating habits and sedentary lifestyle, which could explain the weight gain. Both the girls had normal development of secondary sexual characteristics. Patient T did not attain spontaneous menarche and only had withdrawal bleed once with combined oral contraceptive pills given at the age of 14 years, following which she continued to remain amenorrheic. Patient S had spontaneous menarche at the age of 11 years, followed by amenorrhea for next one year and subsequent resumption of spontaneous but oligomenorrheic menses with intermenstrual interval ranging from 60 to 180 days. Both girls had a history of acneiform eruptions over face and hair loss from scalp, however, there was no history of excessive hair growth over face or other body parts, clitoromegaly, heaviness of voice or breast atrophy. Both the girls had achieved stature as per their expected genetic potential. They denied the history of spontaneous galactorrhea, however, were found to have expressive galactorrhea on examination. There was no history of headache, field defects, acral enlargement, coarsening of facial features or intake of anticonvulsant drugs. Family history was significant for the history of diabetes in mother and maternal grandparents. No history of menstrual irregularities, infertility, or uterine cancer in the family could be elicited. On examination, both girls were found to have central obesity with features of severe IR. Both of them had Tanner stage V breast development, with expressive galactorrhea. Clinical and biochemical findings of the two sisters at baseline have been summarized in Table 1.

**Table 1:** Clinical and biochemical findings of the sisters at baseline. BMI: Body mass index; OGTT: Oral glucose tolerance test; TC: Total cholesterol; TG: Triglyceride; LDL: Low-density lipoprotein cholesterol; HDL: High-density lipoprotein cholesterol; TSH: Thyroid stimulating hormone; DHEAS: Dehydroepiandrosterone sulfate; ONDST: Overnight dexamethasone suppression test; GH: Growth hormone; IGF-1: Insulin-like growth factor 1.

Parameter	Patient T	Patient S
Menstrual cycles	Amenorrhea	Oligomenorrhea
Weight (kg)	81	85
Height (cm)	150	154
BMI (kg/m^2^)	36.0	35.8
Waist circumference (cm)	102	106
Hip circumference (cm)	112	117
Blood pressure (mm Hg)	102/68	102/80
Acanthosis nigricans	Grade IV	Grade IV
Ferriman-Gallewy score	7/36	8/36
Expressive galactorrhea	Present	Present
75 gm OGTT Plasma glucose (0/1/2 hour) (mg/dl)	92/79/101	89/101/91
Serum Insulin (0/1/2 hour) (µIU/ml)	32.23/203.6/132.1	39.27/110.1/91.56
Glycated hemoglobin (%)	5.4	5.2
Fasting lipid profile TC/TG/LDL/HDL (mg/dl)	163/125/100/38	151/232/67/37
SGOT/SGPT (IU/L)	23/28	28/27
Uric acid (mg/dl)	4.8	5.0
T4 (µg/dl)/TSH (µIU/ml) (N: 5.1–14.2/0.27–​​​​​​​4.2)	8.06/2.73	7.76/3.18
8 am Testosterone (ng/ml) (N: 0.08–​​​​​​​0.48)	0.57	0.63
DHEAS (ug/dl) (N: 65–​​​​​​​368)	616.8	737.7
8 am Cortisol (ug/dl)	17.83	27.27
ONDST Cortisol (ug/dl) (N: <1.8)	0.78	0.71
GH (ng/ml)	0.07	0.10
IGF-1 (ng/ml)	166	185
Prolactin (ng/ml) (x2) (N: 6.0–​​​​​​​29.9)	163.0/145.6	112.9/106.3

Both the sisters had biochemical evidence of androgen excess (elevated testosterone and dehydroepiandrosterone sulfate (DHEAS)), IR (elevated homeostatic model assessment of insulin resistance (HOMA-IR)) and hyperprolactinemia. Ultrasonography of the abdomen and pelvis revealed polycystic ovary pattern with normal adrenals in both patients. Magnetic resonance imaging (MRI) of the pituitary gland done to exclude the possibility of sellar or parasellar mass was normal in both.

At this stage, we considered two differential diagnoses. First, an androgen excess disorder related to IR (PCOS) and second, androgen excess related to idiopathic hyperprolactinemia. Since the diagnosis was not clear at this time, we decided to treat the two sisters separately using different agents. Patient T was treated with insulin sensitizer Metformin (1000 mg/day), while patient S was treated with dopamine agonist Cabergoline (0.5 mg/week). Both girls were given advice on dietary and lifestyle modifications to lose weight.

At follow-up visit three months later, patient S resumed regular menses and had resolution of expressive galactorrhea, while patient T continued to remain amenorrheic with expressive galactorrhea. Biochemistry revealed normalization of prolactin, testosterone and DHEAS in patient S, while all these parameters continued to remain high in patient T. While fasting insulin and parameter of IR (HOMA-IR) improved in patient T treated with metformin, it failed to bring normalization of the androgen excess state. Both the girls were briefly lost to follow-up subsequently, only to return at 12 months, when it was decided that cabergoline be initiated in patient T as well. At a recent follow-up visit (18 months), patient T too resumed her menses with biochemical resolution of hyperprolactinemia and androgen excess state. Both the girls have been continued on cabergoline therapy with reinforcement of advice on weight reduction. The serial biochemical investigations have been summarized in Table 2.

**Table 2:** Serial clinical and biochemical findings. DHEAS: Dehydroepiandrosterone sulfate; HOMA-IR: Homeostatic model assessment of insulin resistance.

Parameter	Patient T				Patient S			
	Baseline	Three months (on Metformin)	12 months (stopped Metformin for three months)	18 months (on Cabergoline)	Baseline	Three months (on Cabergoline)	12 month (stopped Cabergoline x 2 months)	18 month (on Cabergoline)
Menstrual Cycles	Amenorrhea	Amenorrhea	Amenorrhea	Resumption of menses	Oligomenorrhea	Regular menses	Oligomenorrhea	Regular menses
Expressive galactorrhea	Present	Present	Present	Absent	Present	Absent	Absent	Absent
8 am Testosterone (ng/ml) (N: 0.08–0.48)	0.57	0.56	0.866	0.282	0.63	0.168	0.41	0.46
DHEAS (ug/dl) (N: 65–​​​​​​368)	616.8	698.4	705.5	156.2	737.7	149.4	-	213.3
HOMA-IR	7.27	4.45	-	4.55	8.66	8.06	-	8.44
Prolactin (ng/ml) (N: 6.0–​​​​​​29.9)	163.0/145.6	130.6	113.6	0.52	112.9/106.3	0.51	134.7	0.30

## Discussion

We have described the case of identical twin sisters who presented to us with features suggestive of marked IR and PCOS. However, on evaluation, they were also found to have hyperprolactinemia, which was confirmed on repeated sampling. There was no drug intake to explain secondary hyperprolactinemia, no clinical or biochemical evidence of acromegaly, and no clinical or radiological evidence of mass lesion in the sellar region. Both hyperprolactinemia and PCOS can cause androgen excess and present with anovulation. While androgen excess in PCOS may be of ovarian or adrenal origin, the hyperandrogenic state in hyperprolactinemia is possibly mediated by increased adrenal androgen production, which improves with dopamine agonist treatment [6,7]. In the case described, both the possibilities of PCOS and idiopathic hyperprolactinemia were initially considered. Subsequently, medications targeting IR and hyperprolactinemia were used separately in the two girls and on the basis of therapeutic response to cabergoline, the girls were diagnosed to have idiopathic hyperprolactinemia.

A bidirectional relationship may exist between hyperprolactinemia and insulin resistant states. In the literature, there are various studies which document elevated levels of prolactin in patients with PCOS [8,9]. An alteration of opiodergic-dopaminergic tone has been the postulated mechanism for this observation. However, recent studies have found that prolactin elevation in these patients is transient and likely related to underlying stress, use of offending drugs or hypothyroidism. In a study by Filho et al., it was found that prolactin levels in women with PCOS were not different from insulin-resistant non-PCOS controls [10]. In the study, women with PCOS phenotype and high prolactin levels had an underlying cause as pituitary adenoma, oral contraceptive treatment or use of offending medication. The authors, therefore, concluded that hyperprolactinemia is not a clinical manifestation of PCOS and, any significant prolactin elevation in patients with suspected PCOS should be investigated further. On the other hand, hyperprolactinemia may itself be associated with IR and induction of glucose intolerance [11]. This is postulated to be related to increased fatty acid concentration, downregulation of insulin receptors and post-binding defect in insulin action. In a study comparing non-obese hyperprolactinemic PCOS patients with age and body mass index (BMI) matched non-obese healthy controls, measures of IR were higher in hyperprolactinemic subjects compared to normoprolactinemic controls, suggesting the role of prolactin in mediating IR [12].

The diagnosis of idiopathic hyperprolactinemia is one of exclusion. It is made after excluding the use of offending drugs, hypothyroidism, acromegaly and any structural lesion on the sellar imaging. It is possible, however, that these patients may have small microadenomas, which may be missed with MRI. Less than 10% of these patients are ultimately found to have microadenoma on serial imaging, while about 30% may have spontaneous normalization of prolactin levels [1]. However, when symptomatic, these patients may be treated with dopamine agonist therapy and withdrawal attempted after two years of follow-up with normal prolactin levels.

## Conclusions

To conclude, we have described a rare association of severe IR, hyperandrogenism, and hyperprolactinemia. Hyperandrogenism was found to be related to hyperprolactinemia and responded with cabergoline therapy. However, since the etiology of hyperprolactinemia remains unknown, a possible role of IR in causation of high prolactin may be considered and needs to be studied further. This report emphasises the importance of excluding alternative disorders of androgen excess before considering a diagnosis of PCOS in routine clinical practice. Any significant and unexplained prolactin elevation in such a scenario should be thoroughly investigated. Hyperprolactinemia may cause androgen excess by increasing adrenal androgen secretion, which resolves on treatment with dopamine agonist cabergoline.
